# Curvature-driven instabilities in thin active shells

**DOI:** 10.1098/rsos.220487

**Published:** 2022-10-12

**Authors:** Andrea Giudici, John S. Biggins

**Affiliations:** Department of Engineering, University of Cambridge, Trumpington Street, Cambridge CB21PZ, UK

**Keywords:** shells, curvature, instability, geometry, morphing

## Abstract

Spontaneous material shape changes, such as swelling, growth or thermal expansion, can be used to trigger dramatic elastic instabilities in thin shells. These instabilities originate in geometric incompatibility between the preferred extrinsic and intrinsic curvature of the shell, which may be modified by active deformations through the thickness and in plane, respectively. Here, we solve the simplest possible model of such instabilities, which assumes the shells are shallow, thin enough to bend but not stretch, and subject to homogeneous preferred curvatures. We consider separately the cases of zero, positive and negative Gauss curvature. We identify two types of supercritical *symmetry-breaking* instability, in which the shell’s principal curvature spontaneously breaks discrete up/down symmetry and continuous planar isotropy. These are then augmented by *inversion* instabilities, in which the shell jumps subcritically between up/down broken symmetry states and *rotation* instabilities, in which the curvatures rotate by 90° between states of broken isotropy without release of energy. Each instability has a thickness-independent threshold value for the preferred extrinsic curvature proportional to the square root of Gauss curvature. Finally, we show that the threshold for the isotropy-breaking instability is the same for deep spherical caps, in good agreement with recently published data.

## 1. Introduction

Elastic structures under load are often susceptible to dramatic instabilities such as the buckling of a column under compression [1,2], or the collapse of a vessel under pressure [3,4]. Traditionally, engineers have studied instabilities to avoid them as they lead to softening, fracture and failure. However, recently, the study of instabilities has been revitalized by the growing interest in soft materials such as gels, elastomers and biological tissues. Soft materials can undergo large strains without failing, meaning they survive instabilities and are thus capable of dramatic shape changes [5]. Furthermore, many soft materials can undergo active strain deformations, including swelling gels [6–10], growth in biological tissues [11–14], uniaxial contraction in liquid crystal elastomers [15–18] and biological muscles, and inflation in barromorphs [19,20] and other pneumatic systems [21]. When these active strains are homogeneous across the material, one obtains simple shape changes. However, when they are inhomogeneous, they are also often geometrically incompatible, meaning that there is no corresponding displacement field that allows the system to fully relax, leading to internal stresses even in the energy-minimizing state.

Such incompatible spontaneous deformations can trigger spontaneous elastic instabilities, in which the system suddenly adopts a new and more complex shape. For example, a growing layer on a soft substrate can wrinkle due to the interfacial mismatch between the length of the layer and the substrate it is attached to [22–26]. Biology uses such growth-induced wrinkling to form brain folds [27,28], villi [13] and gut loops [29]. In thin shell-like structures, incompatible active deformations generate instabilities such as the snap of a Venus fly-trap [30], the folding of a pollen grain [31–33] and the bistability of a cyclist's snap-band [34]. These different shell instabilities have been addressed individually, but a clear and intuitive overview of their behaviour is still lacking. Here, we make a start by presenting a complete and simple treatment of incompatibility-driven instabilities and states in thin and shallow shells, highlighting the different behaviours of positive, zero and negative Gauss curvature geometries.

**Figure 1.**
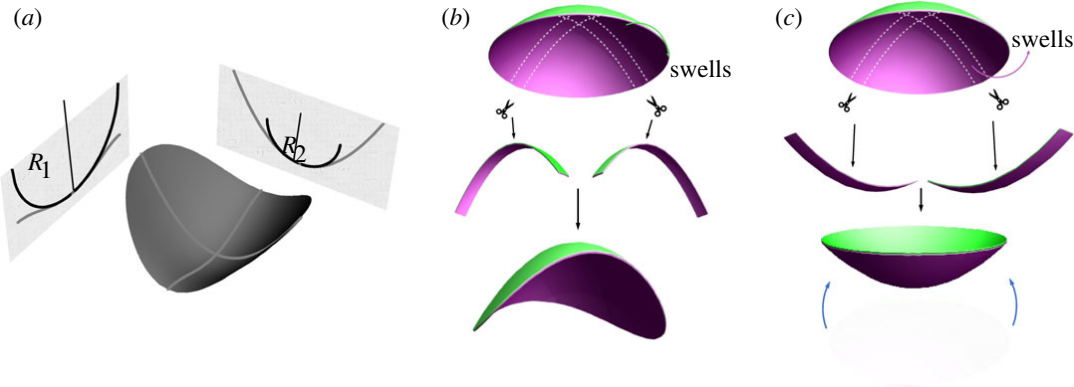
(*a*) Principal curvatures of radius *R*_1_ and *R*_2_ at a point on a shell. (*b*) A spherical cap subject to swelling of the top (green) layer. Cut-out strips want to curve more than what is allowed by the geometry of the spherical cap, leading to the loss of rotational symmetry and folding of the shell. (*c*) A spherical cap subject to swelling of the bottom (purple) layer. Cut-out strips want to curve in the opposite direction as opposed to what is allowed by the geometry of the spherical cap. Excessive opposite bend can lead to snap through inversion of the system (bottom image).

Owing to their slenderness, thin shells can be represented by a two-dimensional surface. Any surface in three-dimensional Euclidean space can be described by its intrinsic and extrinsic properties. The intrinsic geometry is characterized by distances between points on the surface, captured by the metric tensor a. The extrinsic geometry is locally described by the two principal curvatures *κ*_1_ and *κ*_2_, identified by inscribing the largest and smallest possible circles tangent to the surface, as shown in figure 1*a*. Mathematically, aligning the coordinate system with the curvature directions means the curvature is described by the tensor $\kappa =\text{diag}(\kappa_{1},\kappa_{2})$. However, crucially, a and κ cannot be chosen independently. In any physical surface embedded in three-dimensional space, they must satisfy geometric compatibility conditions. Most famously, they must satisfy Gauss’s theorema egregium, stating that the Gauss curvature $K=\text{det}\kappa =\kappa_{1}\kappa_{2}$ is an intrinsic property of the surface and can be computed directly from the metric [35].

In physical shells, active shape changes can separately modify the locally preferred metric $\bar{a}$ by changing in-plane distances [7,9,10,15–19,36,37] and the locally preferred curvature $\bar{\kappa }$ via in-thickness variations, reminiscent of the bending of a bi-metal strip [36,38]. However, given they are controlled by independent mechanisms, it is possible for $\bar{\kappa }$ and $\bar{a}$ to not satisfy the compatibility conditions, meaning that there is no surface that achieves all the preferred characteristics. Such incompatibility between $\bar{\kappa }$ and $\bar{a}$ is the appropriate dimensionally reduced version of general geometric incompatibility in bulk elasticity [39] and results in the physical shell always being internally stressed, even its energy-minimizing state. These internal stresses in turn underpin the instabilities observed in active shells. A key aspect of the mechanics of thin shells is that deforming the metric from $\bar{a}$ requires stretching, with an energetic penalty proportional to thickness *t*, while deforming the extrinsic curvature from $\bar{\kappa }$ only incurs a much smaller bending energy cost proportional to *t*^3^. Thus, in thin shells, isometric bending deformations are favoured wherever possible.

A simple example of incompatibility and instability arises in a spherical bilayer gel cap. If we assume the cap was prepared by casting directly in the spherical state, it will have two equal principal curvatures, 1/*R*, and homogeneous positive Gauss curvature *K* = 1/*R*^2^. These curvatures will also be the preferred values, meaning the cap is compatible and unstressed. The hallmark of this compatibility is that, if we dissect a thin strip from the cap, it will not relax in shape but retain the same curvature it had while part of the cap. However, if we now imagine the upper layer swells mildly (or thermally expands) relative to the lower layer, this will increase the preferred extrinsic curvature beyond 1/*R* while having a negligible effect on the metric. Unfortunately, the shell cannot adopt this increased curvature without also increasing the Gauss curvature, which would require an energetically prohibitive stretch. Instead, it will remain in the same shape but now with internal stresses; if we now dissect out a thin strip, it will be released from its metric constraints and relax to its preferred curvature, as illustrated in figure 1*b*. In this case, further swelling of the top layer ultimately triggers an instability in which the spherical cap loses rotational symmetry and ultimately folds [40], as shown in figure 1*b*. Conversely, large swelling of the bottom layer promotes bends with the opposite sign to the original state, as shown in figure 1*c*. These will eventually drive the snap inversion of the cap, reminiscent of the inversion of a bi-metallic dome used in kettle switches and the snap of a rubber-popper toy [41]. Thus, even in this simple case, we may see two different instabilities, driven by different senses of incompatibility between extrinsic and intrinsic preferred curvature.

Importantly, different instabilities involve different mechanics, as some may proceed purely isometrically, while others require stretching. This difference is reflected in the thickness dependence of the buckling threshold. For example, the folding of the spherical cap subject to a curvature load proceeds via isometries, so the stretching energy is zero and the threshold is thus thickness independent, requiring only a mild excess curvature $\delta \bar{\kappa }=\kappa -\bar{\kappa }∼1/R$. Conversely, the inside-out snapping of the cap inevitably requires stretching and is thus non-isometric. In reality, this instability nucleates at the boundary of the system where bending and stretching compete in a layer of width $∼\sqrt{Rt}$, leading to a much higher buckling threshold that scales like $\delta \bar{\kappa }∼1/\sqrt{Rt}$ [40]. Finally, a fully closed spherical shell has no isometries available and there is no boundary, leading to a yet-higher buckling threshold scaling like $\delta \bar{\kappa }∼1/t$ [40].

By combining casting with initial preferred curvature, gel-like swelling spontaneous shape changes and nematic-elastomer/muscle-like uniaxial spontaneous shape changes, a shell may undergo almost arbitrary programmed changes to $\bar{a}$ and $\bar{\kappa }$ [16–18,36]. In this article, we offer a simple and comprehensive treatment of curvature-driven instabilities in thin, shallow shells with arbitrary but homogeneous preferred curvatures. In such very thin shells, the different scaling of stretching and bending means that the stretching energy turns into a constraint on the metric and hence the Gauss curvature, so one only has instabilities that proceed isometrically, with mild threshold curvature loads $\delta \bar{\kappa }∼\sqrt{K}$. Nevertheless, despite this limitation, the model includes a wide variety of instabilities, in which the principle curvatures of the shell *break-symmetry*, *rotate* and *invert*. The first type of instability is associated with supercritical spontaneous symmetry-breaking and comes in two flavours: the continuous symmetry-breaking of isotropy and discrete breaking of up/down symmetry. The *rotation* instability is a 90° rotation between states of broken isotropy that occurs via a Goldstone-like mode, characterized by a change in configuration but not in energy. Conversely, the *inversion* instability occurs as a subcritical jump between states of broken up/down symmetry and is associated with bistability.

We summarize these results by plotting phase diagrams for active shells with zero, positive and negative Gauss curvature, which show how the state of the shell changes with preferred curvature and where the different instabilities arise. Importantly, our model uncovers new instabilities, especially in the previously little-discussed negative Gauss curvature systems. It also offers a much simpler perspective on several known instabilities that had previously been understood using more-complicated boundary layer physics and/or ad-hoc assumptions. Finally, although our model is for shallow shells, in the final section, we show that the threshold it predicts for the folding of a shallow spherical cap also extends to deep spherical caps.

## 2. Geometric incompatibility and isometric energy

Our task is to find the achieved shape of a shallow and thin shell with a homogeneous preferred curvature $\bar{\kappa }$ and also a preferred metric $\bar{a}$ that, via the theorema egregium, encodes a homogeneous Gauss curvature *K*. Since the shell is thin, we may assume it adopts a final form that exactly achieves $\bar{a}$ and hence bears no stretch energy. Generally, the assumption is always good except near instabilities, where stretching effects may delay the onset by an amount that, however, vanishes as *t* → 0. We will discuss the validity of the no-stretch assumption at several points later in the text.

Furthermore, since the shell is shallow, the achieved surface is approximated by a quadratic form $r(x,y)=(x,y,\frac{1}{2}\kappa_{1}x^{2}+\frac{1}{2}\kappa_{2}y^{2}),$ where *κ*_1_ and *κ*_2_ are the achieved principal curvatures, and (*x*, *y*) are Cartesian coordinates in a tangent plane at the origin, aligned with the curvature tensor $\kappa =\mathrm{diag}(\kappa_{1},\kappa_{2})$. If the preferred curvature is $\bar{\kappa }$, then the bending energy of the shell will be [39]

2.1 $$E_{b}=\frac{Et^{3}}{24(1-\nu^{2})}\int [(1-\nu )\text{Tr}{\left(\kappa -\bar{\kappa }\right)}^{2}+\nu {\left(\text{Tr}\left(\kappa -\bar{\kappa }\right)\right)}^{2}]\;dA.$$

Since the shell bears no stretch energy, the curvature, and thus shape achieved by the shell, corresponds to a minimum of this bend energy. However, the minimization is constrained by the theorema egregium, which dictates that the Gauss curvature *κ*_1_*κ*_2_ of the surface is an intrinsic property of the metric and hence must match *K* as encoded by $\bar{a}$, giving a compatibility constraint between curvature and metric of:

2.2 $$K=\text{det}\kappa =\kappa_{1}\kappa_{2}.$$

Formally, there are two further compatibility equations, known as the Codazzi–Mainardi equations, which also feature the metric connection computed from $\bar{a}$. However, the Cartesian coordinate system (*x*, *y*) forms a normal coordinate system for $\bar{a}$, meaning it is not only orthonormal, but also all the connections vanish at the origin, so the Codazzi–Mainardi equations are automatically satisfied near the origin (and hence throughout our shallow shell) provided κ too is homogeneous, as it is in our quadratic surface. Furthermore, since both κ and $\bar{\kappa }$ are homogeneous, we can trivially evaluate the integral in equation (2.1) to get

2.3 $$E_{b}=\frac{AEt^{3}}{24(1-\nu^{2})}[(1-\nu )\text{Tr}{\left(\kappa -\bar{\kappa }\right)}^{2}+\nu {\left(\text{Tr}\left(\kappa -\bar{\kappa }\right)\right)}^{2}],$$

where *A* is the area of the shell. Therefore, our task is simply to minimize equation (2.3) over κ, subject to equation (2.2). Before we proceed, it is convenient to further simplify the problem by introducing

$$H=\frac{1}{2}\text{Tr}\left(\kappa \right)=\frac{1}{2}(\kappa_{1}+\kappa_{2})\;\text{and}\;D=\frac{1}{2}(\kappa_{1}-\kappa_{2}),$$

which represent the mean achieved curvature and mean difference in achieved curvature of the shell, and the corresponding quantities $\bar{H}=\frac{1}{2}({\bar{\kappa }}_{1}+{\bar{\kappa }}_{2})$ and $\bar{D}=\frac{1}{2}({\bar{\kappa }}_{1}-{\bar{\kappa }}_{2})$ for the preferred curvature. Substituting these into equation (2.3) and rescaling to eliminate the prefactor, we see the problem is equivalent to minimizing

2.4 $$\tilde{E_{b}}=\gamma {\left(H-\bar{H}\right)}^{2}+{\left(D-\bar{D}\right)}^{2}+4D\bar{D}\sin^{2}\theta ,$$

where *θ* is the angle between the principal directions of κ and $\bar{\kappa }$, i.e. it captures the amount of rotation necessary to align the eigenvectors of $\bar{\kappa }$ with those of κ. Moreover, *γ* = (1 + *ν*)/(1 − *ν*) ≥ 1 captures Poisson effects. In what follows, we will always take *γ* = 3 corresponding to *ν* = 0.5 unless stated otherwise. Minimization over κ is now represented by minimization over *H*, *D* and *θ*, while $\bar{D}$ and $\bar{H}$ (preferred curvature) are held constant. Minimization is subject to the reformulated Gauss constraint

2.5 $$K=H^{2}-D^{2},$$

with the Gauss curvature *K* remaining constant as well during minimization. Here, it is important to emphasize that $K\neq \text{det}\bar{\kappa }={\bar{H}}^{2}-{\bar{D}}^{2}$. Once again, this is because in active shape changes, $\bar{a}$ (and hence *K*) and $\bar{\kappa }$ (and hence $\bar{H}$ and $\bar{D}$) can be varied independently, leading to incompatibility. Here, we focus on the case in which $\bar{a}$ and thus *K* do not change, but $\bar{\kappa }$ does.

Before minimizing the energy, we clarify two subtleties in the new parameterization. First, a rotation of the achieved curvature by *θ* = *π*/2 is equivalent to inverting the roles of *κ*_1_ and *κ*_2_, which is also achieved by sending *D* → −*D* without changing *H* and *θ*. Although this might appear as a redundancy, it is helpful to maintain both *D* and *θ* when studying the stability of an equilibrium. Second, flipping the signs of *D*, $\bar{D}$, *H* and $\bar{H}$ leaves the energy unchanged. This is a consequence of the arbitrary definition of positive versus negative curvature. Thus, when studying instabilities, we can often concentrate on a restricted set of values of $\bar{D}$ and $\bar{H}$, then use these facts to extend our theory to all values of the preferred bend.

In the remainder of this paper, we shall discuss this minimization explicitly for the cases when *K* = 0, *K* > 0 and *K* < 0. In each case, we shall present a two-dimensional phase diagram, showing the achieved states of the shell as a function of $\bar{H}$ and $\bar{D}$. Active changes in the shell are then represented by changes in the preferred curvatures, $\bar{H}$ and $\bar{D}$, causing the shell to trace out a path on the phase diagram and instabilities to occur when this line passes from one region to another, indicating a qualitative change in the shape of the shell.

## 3. Instabilities in shallow shells

### 3.1. Gauss flat shells (cylinders)

**Figure 2.**
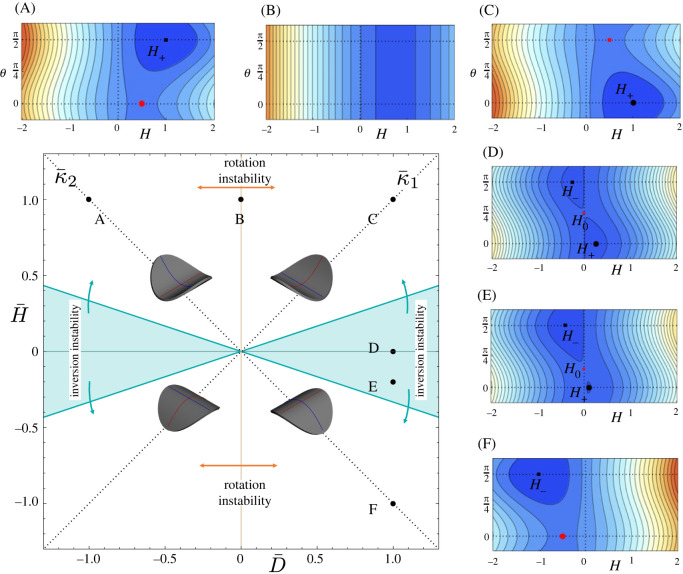
The phase diagram for a Gauss flat system showing various states, accompanied by contour plots of the energy landscape. The stable equilibria (minima) of the energy landscape are depicted by black dots while saddles are depicted by red dots. In the phase diagram, the region where the energy landscape has more than one minimum—the bistable region—is coloured in light blue. Its boundaries are defined by $\left|\bar{D}|>\gamma |\bar{H}\right|$.

We start with the simple case of a flat system that is isometric to the plane, *K* = *κ*_1_*κ*_2_ = 0. The behaviour of such shells is well studied [34,42–46], but we revisit these results in the context of our simple model to clarify their origin and set the context for Gauss-curved systems. The key feature of a Gauss flat sheet is that the Gauss constraint *K* = 0 requires at least one curvature to vanish. Without loss of generality, we take the vanishing curvature to be *κ*_2_, i.e. (*κ*_1_, *κ*_2_) = (*κ*_0_, 0), meaning $D=H=\frac{1}{2}\kappa_{0}$, and the resultant surface is a cylinder. Inserting this Gauss constraint into the bending energy, we find

$$\tilde{E_{b}}=\gamma {\left(H-\bar{H}\right)}^{2}+{\left(H-\bar{D}\right)}^{2}+4H\bar{D}\sin^{2}\theta ,$$

where we have chosen to use *H* rather than *κ*_0_ to describe the magnitude of the curvature for consistency with our later treatment of Gauss-curved systems. Contour plots of the energy as a function of *H* and *θ* are shown in figure 2, for a range of different preferred curvatures (i.e. $\bar{H}$ and $\bar{D}$). We see plots with one or two minima, or even whole lines of minima, which correspond to the stable states of the shell at each preferred curvature. These minima are given by

3.1 $$\frac{\partial \tilde{E_{b}}}{\partial \theta }=4H\bar{D}\sin2\theta =0$$

and

3.2 $$\frac{\partial \tilde{E_{b}}}{\partial H}=2(H-\bar{D}+2\bar{D}\sin^{2}\theta +\gamma (H-\bar{H}))=0.$$

We can also evaluate also the Hessian matrix of second derivatives, which allows us to assess the stability of the solutions of the above equations:

3.3 $$H_{\tilde{E_{b}}}=\left(\begin{array}{cc} 2(1+\gamma ) & 4\bar{D}\sin2\theta \\ 4\bar{D}\sin2\theta & 8H\bar{D}\cos2\theta \end{array}\right).$$

A given equilibrium solution is either a minimum, a saddle or a maximum if the eigenvalues of $H_{\tilde{E_{b}}}$ are positive, mixed or negative, respectively.

#### 3.1.1. Isotropic preferred curvatures, $\bar{D}=0$

The simplest case arises if the preferred curvature is isotropic, meaning the preferred curvature is like a spherical cap with ${\bar{\kappa }}_{1}={\bar{\kappa }}_{2}$ and $\bar{D}=0$. Such a system would arise in a bilayer disc, in which the bottom layer swells or expands relative to the top [42,44]. Of course, this preferred curvature violates the Gauss constraint *K* = 0. However, this does not mean the plate remains flat, as the energy landscape forms a degenerate valley in the *θ* direction at finite $H=\frac{1}{2}\bar{H}(1+\nu )$ [42], as seen in figure 2B. Practically, this means the system chooses to roll up like a cylinder to accommodate the preferred curvature. However, since the preferred curvature is isotropic, all rolling directions are equivalent, and by choosing one, the system breaks a continuous symmetry associated with in-plane isotropy. Within our isometric model, the plate will break symmetry and roll for any finite $\bar{H}$. In reality, a thick plate will initially stretch its mid surface and change *K* to accommodate both preferred curvatures [42], and these symmetry-breaking isometric deformations occur past a threshold that scales like *t*/*R*^2^ [44], which indeed vanishes in the thin limit.

#### 3.1.2. Equal and opposite preferred curvatures, $\bar{H}=0$

A second simple case occurs if the preferred curvatures are equal and opposite. Such a system would arise in a bilayer where the two layers contract uniaxially and equally, but in orthogonal directions, as seen in twisted nematic elastomer sheets [47] or rubber bilayers with orthogonal prestretch [46,48]. In these examples, the spontaneous deformation of the layers is homogeneous across the system and hence does not change the Gauss curvature, while the in-thickness variation alters the preferred curvatures, leading to ${\bar{\kappa }}_{1}=-{\bar{\kappa }}_{2}$. Again, this state of curvature is denied by the Gauss constraint. As shown in figure 2D, the energy landscape now presents two equivalent minima at (*H*, *θ*) = (*H*_+_, 0) and (*H*_−_, *π*/2), separated by a saddle at (0, *π*/4). Practically, this means the system will either roll upwards along the positive preferred curvature or, equivalently, roll downwards along the negative one: the system breaks a discrete up/down symmetry—the two configurations are energetically equivalent—and the system is bistable. As above, in the thin isometric limit, the plate will break symmetry and roll for any finite $\bar{D}$, though, in reality, there will be a small threshold that vanishes with thickness. This bistability has been studied in stiff [43] and soft [45,46] sheets, and is used in cyclist’s snap-bands [34].

#### 3.1.3. General preferred curvatures

At a general point on the phase diagram, away from these two special cases, there may be either one or two minima. Near the $\bar{D}=0$ line, the introduction of a small anisotropy in preferred curvature breaks the degeneracy of the *θ* valley, favouring one end over the other, so that the system is monostable and rolls up into a cylinder along whichever direction has the largest preferred curvature (figure 2A,C). By contrast, near the $\bar{H}=0$ line, the system remains bistable (figure 2E), but the symmetry between the two minima is lost, with the minima corresponding to the larger magnitude of preferred curvature being deeper.

In this *K* = 0 case, we may resolve this behaviour completely analytically. The angular minimization condition, equation (3.1), is everywhere solved by *θ* = 0 and *θ* = *π*/2, meaning the achieved and preferred curvature frames are aligned or anti-aligned. When $\bar{D}<0$ (figure 2A) the minimum at *θ* = *π*/2 corresponds to aligning the principal direction of *κ*_1_ with that of ${\bar{\kappa }}_{2}$ ($>{\bar{\kappa }}_{1}$); while when $\bar{D}>0$ (figure 2C), the minimum at *θ* = 0 corresponds to aligning the principal direction of *κ*_1_ with that of ${\bar{\kappa }}_{1}$ ($>{\bar{\kappa }}_{2}$). Substituting the two values of *θ* in equation (3.2), we find the corresponding *H* values are $H=\frac{1}{2}(1+\nu )\bar{H}+\frac{1}{2}(1-\nu )\bar{D}$ and $H=\frac{1}{2}(1+\nu )\bar{H}-\frac{1}{2}(1-\nu )\bar{D}$, respectively, leading to the actual curvatures [46]

3.4 $$\theta =0,\;\kappa_{0}=\frac{1}{2}({\bar{\kappa }}_{1}+\nu {\bar{\kappa }}_{2})$$

and

3.5 $$\theta =\frac{\pi }{2},\;\kappa_{0}=\frac{1}{2}({\bar{\kappa }}_{2}+\nu {\bar{\kappa }}_{1}).$$

These results show how an orthogonal preferred curvature modifies the achieved curvature via Poisson effects.

In the monostable region, one of these solutions is a saddle and the other is the only minimum, with the assignation delineated by the sign of $\bar{D}$, to align *κ* with the larger preferred curvature. We call the minimum state *H*_+_ if *H* > 0 and *H*_−_ if *H* < 0, as shown in the energy plots of figure 2. This allows us to easily distinguish between rolled up or rolled down cylinders. Mathematically, we have

3.6 $$H=H_{+}\equiv \frac{1}{2}(1+\nu )\bar{H}+\frac{1}{2}(1-\nu )|\bar{D}|$$

and

3.7 $$H=H_{-}\equiv \frac{1}{2}(1+\nu )\bar{H}-\frac{1}{2}(1-\nu )|\bar{D}|.$$

In the bistable region, there is a third solution to equation (3.1), *H* = *H*_0_ = 0, which, via equation (3.2), requires $\theta =\arctan\left(\sqrt{\left(\bar{D}+\gamma \bar{H})/(\bar{D}-\gamma \bar{H}\right)}\right)$. This equilibrium state corresponds to the sheet being flat and only exists in the bistable region $\left|\bar{D}|>\gamma |\bar{H}\right|$, shown in blue on the phase diagram, where both minima *H*_+_ and *H*_−_ coexist, and *H*_0_ forms a saddle between them. As one traverses the bistable region, the *H*_0_ saddle moves between *H*_+_ and *H*_−_, with the collision and resulting merger giving the transition to monostability at the boundary.

#### 3.1.4. Phase diagram and instabilities

The system thus has four basic configurations as shown as insets in the phase diagram, in which either the 1 (*θ* = 0) or 2 (*θ* = *π*/2) direction is curved, either up (*H*_+_) or down (*H*_−_). Each of these configurations is the global minimum in its quadrant and is given by

**Table:** 

*K* = 0	$\bar{D}<0$	$\bar{D}>0$
$\bar{H}>0$	*θ* = *π*/2	*θ* = 0
	*H* = *H*_+_	*H* = *H*_+_
$\bar{H}<0$	*θ* = 0	*θ* = *π*/2
	*H* = *H*_−_	*H* = *H*_−_

Here, intuitively, the sign of $\bar{H}$ is the sign of the preferred mean curvature, where *H*_+_ and *H*_−_ are defined in (3.6). Since the system can roll along one direction up or down, it will choose the curvature of the same sign as the preferred mean curvature. Importantly, each minimum persists as a local minimum above/below its quadrant, within the bistable region where $\left|\bar{D}|>\gamma |\bar{H}\right|$.

Having found the states, we can identify two instabilities. Firstly, if a shell traverses the $\bar{D}=0$ line, as shown by the sequence of states A→B→C, then its direction of achieved curvature will jump by 90° when $\bar{D}=0$. Physically, this occurs when a shell starts with a ${\bar{\kappa }}_{1}>{\bar{\kappa }}_{2}$, but then ${\bar{\kappa }}_{2}$ grows until it first equals and then exceeds ${\bar{\kappa }}_{1}$. In response, the shell rolls into a cylinder, first aligned along ${\bar{\kappa }}_{1}$, then, when the preferred curvature is isotropic $\bar{D}=0$, all rolling directions are degenerate, then after, it rolls along ${\bar{\kappa }}_{2}$. The transition occurs as a rotation of 90° between states of broken isotropy. We call such an instability a *rotation instability*, as the frame of achieved curvature rotates. Curiously, as the rotation occurs along a degenerate valley, no energy is released, although the configuration of the shell does change in a subcritical way. This instability does not seem to have previously been discussed theoretically, but it was recently observed experimentally (using a bilayer structure composed of a passive and singly curved PET shell and a contractile nematic elastomer layer) and deployed for robotic locomotion [49].

The second instability arises when we traverse through the bistable region, resulting in the shell inverting subcritically by jumping between the two bistable states, as shown in the sequence C→D→E→F. Physically, we start with a shell with ${\bar{\kappa }}_{1}>0$, ${\bar{\kappa }}_{2}=0$, so that the shell starts in a monostable region with a single minimum at *H*_+_ (C), corresponding to rolling along ${\bar{\kappa }}_{1}$. If ${\bar{\kappa }}_{2}$ then becomes increasingly negative, the shell will enter the bistable region, with a new local minimum *H*_−_ (rolling along ${\bar{\kappa }}_{2}$) and a saddle between them at *H*_0_. However, the shell will remain stuck rolled along $\bar{\kappa_{1}}$, even as we pass the $\bar{H}=0$ line, and the new minimum becomes the global one. As the shell traverses the bistable region, the *H*_+_ minimum moves towards *H* = 0 due to Poisson effects and, finally, merges with *H*_0_ = 0 to become a saddle, so the shell can jump, subcritically, to *H*_−_ and roll negatively along $\bar{\kappa_{2}}$. This jump occurs between states of broken up/down symmetry and is thus an *inversion instability*, which swaps both the direction and the sign of its achieved curvature. The threshold for instability is

3.8 $$\bar{H}=-\frac{\bar{D}}{\gamma }\;\;⟺\;\;{\bar{\kappa }}_{2}=-\frac{{\bar{\kappa }}_{1}}{\nu }$$

and the instability is a true subcritical instability, in which the configuration of the shell jumps and a finite amount of energy is released.

The inversion instability of *K* = 0 systems has been studied in [45,46] but with results slightly different to those presented here. In the first case, an isometric model was considered, as here, but the instability threshold was established by equating the energies of the two configurations, rather than at the limit of bistability. Conversely, in the latter work, the instability was addressed by studying the boundary layer in which curvature and stretch compete, leading to a thickness-dependent threshold: this is surely important in thick systems, but the isometric model should suffice for thin systems and clarifies that the key physics of inversion arise isometrically in the bulk of the plate, rather than at its boundary.

Finally, we remark about the scale invariance of the Gauss flat system. This invariance arises when assuming the system is thin, Gauss flat and shallow, and we ignore the boundaries. All these assumptions remove any length scale from the problem. Thus, all that is needed to predict the behaviour of the shell is the relative magnitude of $\bar{H}$ and $\bar{D}$, leading to the phase diagram being delineated by straight lines.

### 3.2. Positive Gauss curvatures

Let us now discuss shells with positive Gauss curvature in which the two principal curvatures must have the same sign to satisfy *K* = *κ*_1_*κ*_2_ > 0. We now chose to solve the Gauss constraint in equation (2.5) for *H* and obtain $H=\pm \sqrt{K+D^{2}}$, so that the resultant energy is now a function of *D* (and *θ*), which is the natural order parameter for the cap-folding instability highlighted in the §1. This choice of the sign of *H* corresponds to the shell breaking the discrete up/down symmetry of the flat state as a consequence of its metric: all *K* > 0 shells must break this symmetry. The choice of sign describes two different sets of states. The positive sign corresponds to the set of positive curvatures, where *κ*_1_, *κ*_2_ > 0 and therefore $H>\sqrt{K}$, while the negative sign corresponds to a set of inverted states with *κ*_1_, *κ*_2_ < 0, $H<-\sqrt{K}$. Importantly, unlike in the flat case, there are no isometric paths between these two sets, as any such path would have to pass a point where at least one curvature vanished, giving *K* = 0 and violating the Gauss constraint. Thus, the two sets are disconnected and can be treated separately.

From the bending energy of the shell

**Figure 3.**
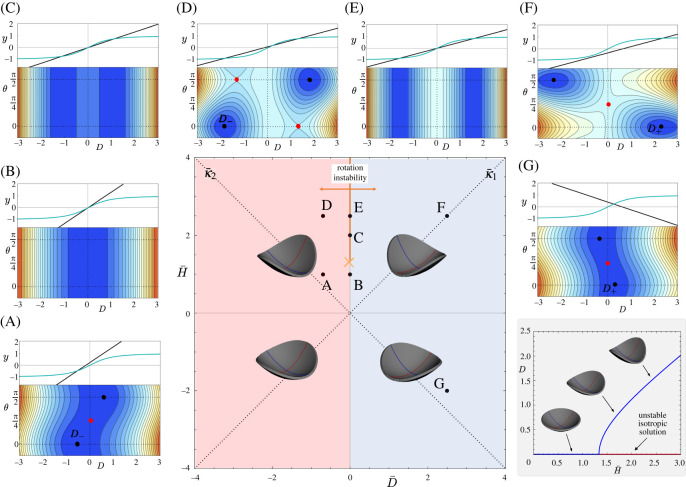
At the centre of the figure, we show the phase diagram for a system with positive Gauss curvature. Around it, the contour plots of the energy for various states (A to G). Above each energy plot, we show the intersection between the two lines in equation (3.15), which yield equilibrium solutions in the case *θ* = 0. Note that all contour plots have the symmetry *D* → −*D* and *θ* → *θ* + *π*/2. Once again, we identify stable equilibria (minima) via black dots and saddle points via red dots, with the *D* = 0, *θ* = *π*/4 equilibrium being always a saddle point. Finally, on the bottom right we show how the amplitude of the symmetry-breaking instability behaves as a function of $\bar{H}$.

3.9 $$\tilde{E_{b}}=\gamma {\left(\pm \sqrt{K+D^{2}}-\bar{H}\right)}^{2}+{\left(D-\bar{D}\right)}^{2}+4D\bar{D}\sin^{2}\theta ,$$

we note that changing the sign of $\sqrt{K+D^{2}}$ (and therefore choosing the other set of states) or changing the sign of $\bar{H}$ both lead to the same energy. This means that results for one set of states can be mapped to the other set by simply flipping the signs of both *H* and $\bar{H}$. We therefore fix *K* = 1, equivalent to rescaling every curvature by $\sqrt{K}$, and consider the positive set of states with $H=\sqrt{D^{2}+1}>0$. Contour plots of the energy as a function of *D* and *θ* are shown in figure 3, for a range of different preferred curvatures, again showing states with different patterns of minima. Importantly, the physical state of the cap is unchanged if we flip *D* → −*D* and send *θ* → *θ* ± *π*/2, so each physical state of the cap appears twice on each plot, once with *D* positive and once with *D* negative. The equilibrium states can be found by minimizing this energy with reference to variations in *θ* and *D*, leading to

3.10 $$D\bar{D}\sin2\theta =0$$

and

3.11 $$D\left(\frac{1+\gamma }{\bar{H}\gamma }\right)-\frac{\bar{D}}{\bar{H}\gamma }\cos2\theta =\frac{D}{\sqrt{1+D^{2}}}.$$

We may also compute the Hessian matrix of second derivatives to characterize the stability of solutions, as follows:

3.12 $$H_{\tilde{E_{b}}}=\left(\begin{array}{cc} 2+2\gamma \left(1-\frac{\bar{H}}{{\left(1+D^{2}\right)}^{3/2}}\right) & 4\bar{D}\sin2\theta \\ 4\bar{D}\sin2\theta & 8D\bar{D}\cos2\theta \end{array}\right).$$

#### 3.2.1. Isotropic preferred curvatures, $\bar{D}=0$

Again, the simplest case arises when the preferred curvature is isotropic, ${\bar{\kappa }}_{1}={\bar{\kappa }}_{2}=\bar{H}$ and $\bar{D}=0$. Physically, this is the case of the bilayer spherical cap, in which one layer swells relative to the other [40]. The isotropic preferred curvature means any *θ* dependence in the energy is lost, $\tilde{E_{b}}=\gamma {\left(\sqrt{1+D^{2}}-\bar{H}\right)}^{2}+D^{2}$. Naively, one might think the system chooses an isotropic state (*D* = 0) to maintain the rotational symmetry. From state *B* in figure 3, we note this is true when the preferred isotropic curvature $\bar{H}$ is smaller than a critical value given by

3.13 $${\bar{H}}_{c}=\frac{2}{1+\nu },$$

leading to an energy landscape with a degenerate minimum valley at *D* = 0, corresponding to the rotationally symmetric isotropic state. We call this state the *D*_0_ state. However, as shown in state *C* in figure 3, when the isotropic preferred curvature is large, $\bar{H}>{\bar{H}}_{c}$, *D*_0_ becomes a maximum ridge and two new minima with $D_{\pm }=\pm \frac{1}{2}\sqrt{{\bar{H}}^{2}{\left(1+\nu \right)}^{2}-4}$ emerge, creating an energy landscape with two degenerate valleys at finite *D*. The two valleys each describe the same set of physical states, which are states in which the cap has broken symmetry and is folded, having chosen to accommodate the larger preferred curvature in one direction, at the cost of even less curvature in the orthogonal direction to maintain *K*. Since the preferred curvature is isotropic, the larger curvature may fold the shell in any direction. However, as in the flat case, by choosing a folding direction the shell breaks a continuous symmetry. This time the preferred curvature threshold is non-vanishing and arises when the preferred curvature is larger than that allowed by the Gauss constraint, meaning the transition can be observed by varying the curvature with a post-buckling scaling $|D|∼\sqrt{\bar{H}-{\bar{H}}_{c}}$, as shown at the bottom right of figure 3. Interestingly, weaker and negative preferred curvatures do not lead to an analogous instability, and the shell conserves its shape.

A graphical perspective can help understand this instability. Since equation (3.10) is automatically satisfied when $\bar{D}=0$, the details of the instability are all contained in equation (3.11), which becomes

3.14 $$D\left(\frac{1+\gamma }{\bar{H}\gamma }\right)=\frac{D}{\sqrt{1+D^{2}}}\;$$

and must be solved for *D*. The function on the left-hand side is a straight line with gradient $(1+\gamma )/\bar{H}\gamma$, while on the right-hand side we have a sigmoidal function which smoothly transitions from −1 to 1, and solutions are intercepts between these two graphs. Examples are shown above the energy plots in figure 3. Increasing $\bar{H}$ makes the gradient of the straight line shallower, causing a transition from one intersection at *D* = 0 (state B) to three intersections at *D* = *D*_−_, 0, *D*_+_ above the critical value *H*_*c*_ (states C and E).

This instability has been observed experimentally in spherical caps made of bilayer swelling gels [40]. However, the loss of symmetry was attributed to the competition between bend and stretch in the boundary layer. Our simple model shows that the same instability is predicted by isometric deformations of the bulk, again offering a simpler perspective on the phenomena.

#### 3.2.2. General preferred curvatures

If the preferred curvature is anisotropic, $\bar{D}\neq 0$, the symmetry of the preferred curvatures is broken, meaning one principal preferred curvature is larger than the other. In this case, equation (3.10) is solved by *θ* = 0, *π*/2, indicating the achieved curvature will align or anti-align with the preferred curvatures.

Indeed, if we start from the isotropic case with $\bar{H}<{\bar{H}}_{c}$ and $\bar{D}=0$ (state B) and then introduce a small asymmetry in the preferred curvature by increasing $\bar{D}$, the result is to both skew the degenerate valley and break its degeneracy (state A) so that it has (physically equivalent) minima at *θ* = 0, *π*/2 separated by a saddle at *D* = 0, *θ* = *π*/4. The system is thus monostable, with an asymmetric achieved curvature that mirrors the preferred one. For the *θ* = 0 state, (3.10) becomes

3.15 $$D\left(\frac{1+\gamma }{\bar{H}\gamma }\right)-\frac{\bar{D}}{\bar{H}\gamma }=\frac{D}{\sqrt{1+D^{2}}},$$

and the solution gives the minimizing value of *D*. In analogy with the flat case, we call the solution *D*_−_ if it is a minimum of the energy with *D* > 0 and *D*_+_ for a minimum with *D* > 0. Although this equation does not admit analytic solutions, we see that this is still the intersection of a line and a sigmoidal function, and the effect of $\bar{D}$ is simply a vertical offset in the line, moving the solution away from the origin.

Conversely, if we start from an isotropic preferred curvature with $\bar{H}>{\bar{H}}_{c}$, we start with a landscape with two degenerate valleys (state C), describing folded-cap states, where the achieved curvature is already asymmetric but may align in any direction. Now, the introduction of asymmetry in preferred curvature breaks the degeneracy of the valley, so that it has a minimum at one end, corresponding to the fold being aligned with the greater preferred curvature, and a saddle at the other, with anti-alignment (state D). The two valleys still describe the same set of physical states, so the two minima in plot D are the same physical state and the shell is still monostable. An analytical solution for *D* can be found via the substitution *D* = sin*ψ*, yielding a quadratic equation for e^*ψ*^. However, this approach is cumbersome and not very informative. A better alternative is to find the *θ* = 0 solution for *D* graphically by considering equation (3.15): the straight line is offset vertically, but there are still three intersections corresponding to a minimum (left), maximum (middle) and saddle (right), as shown in plot D. The physically equivalent solutions for *θ* = *π*/2 could be obtained from an analogous graphical method.

As we further increase $\bar{D}$, and hence the vertical offset of the line, we will ultimately transition from having three intersections to having one, recovering a landscape like state F, with two minima and a saddle. The transition between these two types of landscapes occurs at ${\bar{D}}^{2}={\left(\sqrt[{3}]{2{\left(\nu +1\right)}^{2}{\bar{H}}^{2}}-2\right)}^{3}/2{\left(1-\nu \right)}^{2}$. When *D* is smaller than this critical value, a 1D cut of the energy along the *θ* = 0 line appears double-welled, while after the transition above this critical value it appears single welled. However, one must not conclude that this is therefore a transition from bistable to monostable, as, in every case, the subsidiary minimum in the *D* direction is unstable in the angular direction and hence a saddle overall. In reality, the system is monostable for every finite $\bar{D}$.

#### 3.2.3. Phase diagram and instabilities

Bringing this all together, we may summarize the stable states of the spherical cap as

**Table:** 

*K* = 1	$\bar{D}<0$	$\bar{D}>0$
*H* > 0	$D=D_{-}(\bar{H},\bar{D})$	$D=D_{+}(\bar{H},\bar{D})$
	$H=\sqrt{1+D^{2}}$	$H=\sqrt{1+D^{2}}$
	*θ* = 0	*θ* = 0
*K* = 1	$\bar{D}<0$	$\bar{D}>0$
*H* < 0	$D=D_{-}(-\bar{H})$	$D=D_{+}(-\bar{H})$
	$H=-\sqrt{1+D^{2}}$	$H=-\sqrt{1+D^{2}}$
	*θ* = 0	*θ* = 0

where the table shows the two disconnected sets of states with *H* > 0 (top) and *H* < 0 (bottom), and we have not explicitly included $\bar{H}$ in the table since changing its sign does not affect the nature of the solution. Importantly, when $\bar{D}<0$, we have that $D_{-}(\bar{H})<0$ is the left-most root of equation (3.15), while $D_{+}(\bar{H})>0$ is the right-most root. Conversely, if $\bar{D}>0$, the intersections are instead saddle (left), maximum (middle) and minimum (right). Hence, the energy maximum approaches the saddle point in either case as $\left|\bar{D}\right|$ increases. The values of the minima of the inverted set of states can be thought of in two ways. The straightforward way is to consider the other sign of *H*, meaning *H* < 0 is the inverted cap. However, this cap can be turned upside down, meaning *H* > 0 once again, but now $\bar{H}$ has changed sign. Thus, minima of the inverted states can be found by replacing $\bar{H}$ with $-\bar{H}$ in the equations for the original spherical cap, as shown in the table.

As illustrated in figure 3, across *D* = 0, the *D*_+_ and *D*_−_ states merge continuously via *D* = 0 below ${\bar{H}}_{c}$ but are discontinuous above ${\bar{H}}_{c}$, where they are equal and opposite. This feature leads to two instabilities. Firstly, as already discussed, if we have isotropic but increasing preferred curvature, $\bar{D}=0$, $\bar{H}>0$, then the shell remains isotropic for *H* < *H*_*c*_ but breaks isotropy and folds in a *continuous symmetry-breaking* instability for *H* > *H*_*c*_. This is the trajectory B −> C −> E on the diagram and leads to a supercritical growth of *D* beyond *H*_*c*_, as shown in the bottom right of figure 3. Secondly, if the shell crosses the $\bar{D}=0$ line above *H*_*c*_, D→E→F, it will jump discontinuously between states of broken isotropy, from *D*_−_ to *D*_+_. At the moment of discontinuity, the sign of *D* simply flips, so this is a *rotation instability*, in which the direction of greater folding rotates by 90° so that it always matches the larger preferred curvature. As in the Gauss flat state, it produces a finite change in the configuration of the shell but no release of energy.

Our isometric model does not allow transitions between the two disconnected sets of states, which would be *inversion instabilities*. In reality, such instabilities do occur, but via non-isometric pathways and with the stability threshold diverging for very thin shells [40,50].

### 3.3. Negative Gauss-curved surfaces

Finally, we look at shells with negative Gauss curvature, *κ*_1_*κ*_2_ = *K* < 0, in which the two principal achieved curvatures must have opposite signs forming a saddle. The metric thus requires a choice of orientation of the principal direction, meaning that all *K* < 0 shells break the isotropy of the flat state. To establish the bending energy, we first solve the Gauss constraint in equation (2.5). As we have done in the flat case, we return to solving for *D*, obtaining $D=\pm \sqrt{H^{2}-K}$. Without loss of generality, we set *K* = −1 and select the positive solution $D=\sqrt{1+H^{2}}$, leading to the bending energy

3.16 $$\tilde{E_{b}}=\gamma {\left(H-\bar{H}\right)}^{2}+{\left(\sqrt{H^{2}+1}-\bar{D}\right)}^{2}+4\sqrt{H^{2}+1}\bar{D}\sin^{2}\theta .$$

**Figure 4.**
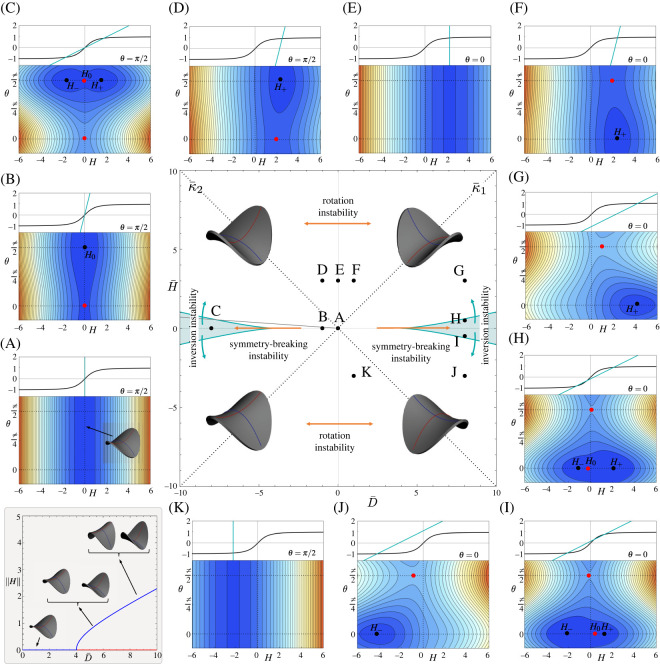
At the centre, the phase diagram for a system with negative Gauss curvature. Around it, the energy contours of different states (A to K are shown). Above each energy plot, we show the two lines appearing on either side of equation (3.18). Their intersection coincides with the minima and saddle point in the energy landscape, shown by black (stable) dots and red (unstable) dots in the contour plots. Once again, the region where two stable solutions exist—the bistable region—is shaded in light blue. Its boundaries are described in equation (3.23). On the bottom left, we show a plot of the amplitude of the symmetry-breaking instability as a function of $\bar{D}$.

Again, this energy is plotted for a range of preferred curvatures in figure 4. The equilibrium states of the system can be found by minimizing this energy with reference to variations in *θ* and *H*, leading to the equations

3.17 $$\bar{D}\sqrt{1+H^{2}}\sin2\theta =0\;$$

and

3.18 $$\frac{H(1+\gamma )}{\bar{D}\cos2\theta }-\frac{\bar{H}\gamma }{\bar{D}\cos2\theta }=\frac{H}{\sqrt{1+H^{2}}}.$$

The first equation is solved by *θ* = 0 or *θ* = *π*/2, indicating the bends are aligned or anti-aligned with the preferred ones, or by $\bar{D}=0$, indicating the preferred curvature is isotropic, so the energy is insensitive to orientation. The second equation contains much more complexity and, similar to the positive Gauss curvature case, is composed of a straight line on the left-hand side and a sigmoidal function on the right-hand side. However, in this case, the main variable is *H* and the offset depends on $\bar{H}$.

Finally, we also evaluate the Hessian matrix that will allow us to discuss the stability of equilibrium solutions

3.19 $$H_{\tilde{E_{b}}}=\left(\begin{array}{cc} 2\left(1+\gamma -\frac{\bar{D}\cos2\theta }{{\left(1+H^{2}\right)}^{3/2}}\right) & \frac{4\bar{D}H\sin2\theta }{\sqrt{1+H^{2}}}\\ \frac{4\bar{D}H\sin2\theta }{\sqrt{1+H^{2}}} & 8\bar{D}\sqrt{1+H^{2}}\cos2\theta \end{array}\right).$$

#### 3.3.1. Zero preferred curvatures, $\bar{H}=\bar{D}=0$

We first consider a shell with zero (flat) preferred curvature, i.e. $\bar{D}=\bar{H}=0$. In this case, a positive or flat Gauss shell achieves a state that mirrors the preferred rotational symmetry by taking the isotropic spherical cap or fully flat state, respectively. However, a negative Gauss surface must form a saddle shape and therefore cannot maintain the rotational symmetry. This key difference is what characterizes much of the behaviour of negative surfaces. For the negative *K* case, we obtain an energy landscape of the state like A in figure 4, with a degenerate valley at *H* = 0 (and hence *D* = 1) corresponding to a state in which the two principal curvatures are equal in magnitude (*κ*_1_ = −*κ*_2_ = 1, *H* = 0) but in any orientation. We call such a state with equal magnitude curvatures a *symmetric* saddle shape.

#### 3.3.2. Isotropic preferred curvatures, $\bar{D}=0$

We now turn to isotropic but non-zero preferred curvature, meaning ${\bar{\kappa }}_{1}={\bar{\kappa }}_{2}$, $\bar{D}=0$ and $\bar{H}\neq 0$. An energy plot for $\bar{H}>0$ is shown in state E of figure 4, clearly highlighting a valley at $H=\frac{1}{2}\bar{H}(1+\nu )>0$, which, naturally, is degenerate in *θ*. These states are asymmetric saddles, with the larger magnitude curvature having the same sign as the preferred curvature. However, since the preferred curvature is isotropic, the achieved asymmetric saddle can be oriented along any direction.

#### 3.3.3. Symmetric preferred saddle, $\bar{H}=0$

There is another regime, important in systems of negative Gauss curvature, in which the preferred curvature itself is a symmetric saddle, meaning the two preferred curvatures have equal magnitudes but opposite sign, ${\bar{\kappa }}_{1}=-{\bar{\kappa }}_{2}$ ($\bar{H}=0$ and $\bar{D}\neq 0$), giving the energy a discrete up/down symmetry. Naively, we may think this preferred curvature always favours a symmetric saddle (*H* = 0) with matching curvatures, i.e. aligning the positive curvature with the preferred positive curvature and the negative with the preferred negative. As shown by the energy plot of state B in figure 4, this is true when the preferred saddle is weak, since the energy landscape for $\bar{D}<0$ forms a valley about *H* = 0 with a minimum at *θ* = *π*/2 and a saddle solution at *θ* = 0. The minimum and the saddle correspond to the achieved saddle being aligned and anti-aligned with the preferred one.

However, as shown in state C of figure 4, when the curvature load $\left|\bar{D}\right|$ is larger than a critical value

3.20 $${\bar{D}}_{c}=\frac{2}{1-\nu },$$

the minimum in the valley splits into two symmetric minima at $H_{\pm }=\pm \frac{1}{2}\sqrt{{\bar{D}}^{2}{\left(1-\nu \right)}^{2}-4}$ and *θ* = *π*/2. The two minima give rise to a bistable region, reflecting a preference of the system to partially accommodate either one of the two large preferred principal curvatures rather than remaining symmetric and accommodating neither. The same behaviour is observed when $\bar{D}>0$ and $\bar{D}>{\bar{D}}_{c}$, with a minimum at *θ* = 0 instead. The choice of which curvature is accommodated is associated with the breaking of the energy’s discrete up/down symmetry. As shown in the inset on the bottom left of figure 4, this behaviour is a supercritical, leading to a post-buckling amplitude scaling, $|H|∼\sqrt{\bar{D}-{\bar{D}}_{c}}$.

Curiously, the mathematics of this discrete symmetry-breaking transition with *K* < 0 are very similar to the continuous symmetry-breaking for *K* > 0. As in the positive case, it is again helpful to consider the situation graphically. Without loss of generality, we take $\bar{H}=0$, $\bar{D}<0$, like state C in figure 4. Equation (3.17) is solved by *θ* = 0 and *θ* = *π*/2. To find the minima, we first look at the correctly matched saddle, with *θ* = *π*/2. Then, equation (3.18) becomes

3.21 $$-H\frac{\left(1+\gamma \right)}{\bar{D}}=\frac{H}{\sqrt{1+H^{2}}}.$$

This structure is indeed familiar from the positive Gauss surface, equation (3.14), where the gradient of the line determines the number of solutions. When $\left|\bar{D}\right|$ is small, we have only one solution, which we call *H*_0_ and corresponds to the symmetric saddle with *H* = 0. When $|\bar{D}|>{\bar{D}}_{c}$, the line intersects the sigmoidal function two more times generating solutions $H_{\pm }=\pm \frac{1}{2}\sqrt{{\bar{D}}^{2}{\left(1-\nu \right)}^{2}-4}$. These two solutions are minima, while the *H*_0_ solution becomes a maximum as can be checked from the Hessian matrix (3.18).

When $\bar{D}<0$, the state with *θ* = 0 corresponds to a saddle rotated the wrong way around and with mismatched curvatures. In this case, the second equilibrium condition equation (3.18) becomes $H(\left(1+\gamma \right)/\bar{D})=H/\sqrt{1+H^{2}}$ and has only solution, *H* = 0, a symmetric saddle point with the curvatures aligned the wrong way around. This state is obviously unstable, as can be easily checked from the Hessian matrix; the system wants to rotate to align its bends correctly. When $\bar{D}>0$, the *θ* = 0 solution becomes the minimum, while the *θ* = *π*/2 becomes the saddle point.

#### 3.3.4. General preferred curvatures

We will discuss what happens if we move a little away from the isotropic preferred curvature $\bar{D}=0$ in the phase diagram (state E) by introducing a small anisotropy to the preferred curvature. In this case, the effect is to break the angular degeneracy, leading to a state like F in which there is a single minimum, corresponding to an asymmetric achieved saddle with a determined alignment. It is also helpful to consider stepping away from the symmetric preferred saddle shape with $\bar{H}=0$, by introducing a small asymmetry in the magnitudes of the preferred curvature. If we start from a monostable state like B with |*D*| < *D*_*c*_, with a single symmetric-saddle minimum at *H* = 0, then the small asymmetry simply moves this minimum away from the origin, as in state D, leading to a monostable landscape in which the shell adopts a mildly asymmetric saddle reflecting the now asymmetric preferred curvatures. By contrast, if we start from a bistable situation, like C, with |*D*| > *D*_*c*_, then asymmetry initially breaks the symmetry between the two minima, which become a global minimum where the achieved asymmetry is aligned with the preferred curvature and a local minimum where it is anti-aligned, as seen in state H. It follows that there is a region of bistability on the phase diagram and a region of monostability.

The values of *H* for these general states can be found analytically, but the solutions are cumbersome and not very informative. Once again, it is better to consider a graphical approach. If we focus on $\bar{D}>0$ (the right side of figure 4), the minima are found at *θ* = 0, which solve (3.16). The second equilibrium condition takes the form

3.22 $$\frac{H(1+\gamma )}{\bar{D}}-\frac{\bar{H}\gamma }{\bar{D}}=\frac{H}{\sqrt{1+H^{2}}},$$

which is once more the intersection of an offset line with the sigmoidal function. As seen in figure 4, the set of solutions are exactly analogous to those discussed for the positive case, equation (3.15), with the system having only one solution when $\bar{D}$ is small but three solutions when $\bar{D}$ is large. In the region with one solution, we call the minimum *H*_+_ or *H*_−_ depending on whether its *H* value is greater or less than zero. Since there is only one minimum, the system is monostable. When there are three solutions, we classify the three roots, moving from left to right, as *H*_−_ < 0, *H*_0_ and *H*_+_ > 0. The states *H*_−_ and *H*_+_ are still minima, which now coexist, and *H*_0_ is a saddle point, meaning the system is bistable. The global minimum is the root with the same sign as $\bar{H}$. An exactly analogous situation arises for $\bar{D}<0$ but with *θ* = *π*/2.

At the limit of bistability, the top-right entry of the Hessian matrix vanishes. This observation allows us to find the limit of the bistable region as

3.23 $${\bar{H}}^{2}=\frac{{\left({\;}^{3}\sqrt{2{\left(1-\nu \right)}^{2}{\bar{D}}^{2}}-2\right)}^{3}}{2{\left(\nu +1\right)}^{2}}.$$

#### 3.3.5. Phase diagram and instabilities

We now can discuss all the (minimum energy) states in the system. First, we note that there are four main states, covering the combinations of the principal achieved curvature signs and magnitude asymmetries. These four states are exemplified by the insets in the four quadrants of the phase diagram in figure 4 and are each the global minimum within their quadrant

**Table:** 

*K* = −1	$\bar{D}<0$	$\bar{D}>0$
$\bar{H}>0$	*θ* = *π*/2	*θ* = 0
	*H* = *H*_+_	*H* = *H*_+_
	$D=\frac{1}{2}\sqrt{1+H_{+}^{2}}$	$D=\frac{1}{2}\sqrt{1+H_{+}^{2}}$
$\bar{H}<0$	*θ* = *π*/2	*θ* = 0
	*H* = *H*_−_	*H* = *H*_−_
	$D=\frac{1}{2}\sqrt{1+H_{-}^{2}}$	$D=\frac{1}{2}\sqrt{1+H_{-}^{2}}$

where *H*_+_ > 0 and *H*_−_ < 0 are the right- and left-most roots of (3.21). Furthermore, each state persists as a local minimum above/below its quadrant, within the region of bistability. Importantly, *H*_+_ and *H*_−_ connect continuously via *H* = 0 (a symmetric achieved saddle) across $\bar{H}=0$ for $|\bar{D}|<{\bar{D}}_{c}$ but discontinuously for $|\bar{D}|>{\bar{D}}_{c}$ in the bistable region.

The negative Gauss shell can thus sustain three types of instability. All *K* < 0 saddles break the isotropy of a plane, even in the isotropic preferred case $\bar{D}=0$. Crossing the $\bar{D}=0$ line, D→E→F, thus leads to a *rotation instability*, where the (asymmetric) achieved saddle will rotate through states of broken isotropy by 90°, to conform with the preferred anisotropy. Conversely, a saddle does not by default break up/down symmetry, but moving along the $\bar{H}=0$ line eventually triggers a supercritical *symmetry-breaking instability* at ${\bar{D}}_{c}$, in which a symmetric saddle becomes a bistable asymmetric saddle, with one curvature larger in magnitude than the other, B→C, and broken up/down symmetry. Crossing through the resulting bistable region by varying $\bar{H}$, path G→H→I→J, will give a sub-critical *inversion instability* where the shell jumps from *H*_+_ to *H*_−_ between states of broken up/down symmetry at the limit of bistability shown in equation (3.23). During inversion, the saddle exchanges the asymmetry of its curvatures without exchanging their signs.

## 4. Curvature-driven loss of stability in deep spherical caps

As discussed in §3.2.1, a shallow spherical cap subject to an excess preferred curvature loses rotational symmetry and folds when the preferred curvature exceeds a critical value ${\bar{\kappa }}_{c}=(2/(1+\nu ))\sqrt{K}$, promoting the folding of the structure. In this section, we show that the critical buckling load and the twofold symmetric buckled shape predicted by the simple shallow model are correct even in deep shells, highlighting the shallow model’s value.

**Figure 5.**
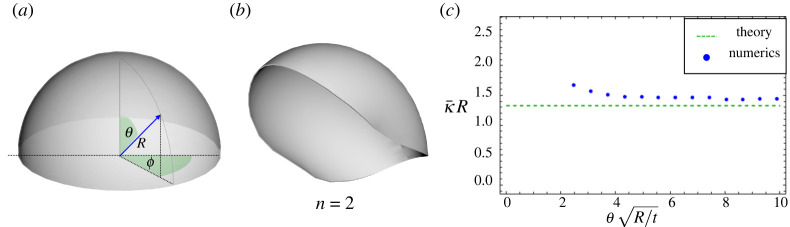
(*a*) Schematics of the coordinate system used to describe a deep spherical cap. The cap has depth *θ*_0_. (*b*) The folding deformation associated with the mode *n* = 2 of the Rayleigh isometry. (*c*) Comparison between numerical simulations and theoretical prediction of the buckling threshold inducing the loss of rotational symmetry in a spherical cap as a function of the cap depth $\bar{\theta }=\sqrt{R/t}\theta_{0}$. Numerics (blue points) for spherical caps of thickness *t*/*R* = 10^−3^ are taken from [40], while our buckling threshold (dashed green line) is $\bar{\kappa }R=2/(1+\nu )$.

We start by considering a spherical shell with angular depth *θ*_0_, and described with spherical coordinates (*R*, *θ*, *ϕ*), as shown in figure 5*a*. We take an isotropic preferred curvature $\bar{\kappa }=\bar{\kappa }I$, as would result in a bilayer gel shell, and, without loss of generality, we set *K* = 1. Following our work for the shallow shell, let us write the principal curvatures as

4.1 $$\kappa_{1}(\theta ,\phi )=H(\theta ,\phi )+D(\theta ,\phi )$$

and

4.2 $$\kappa_{2}(\theta ,\phi )=H(\theta ,\phi )-D(\theta ,\phi ),$$

where $H=\frac{1}{2}(\kappa_{1}+\kappa_{2})>0$ is the mean curvature and $D=\frac{1}{2}(\kappa_{1}-\kappa_{2})$ is the curvature asymmetry. Note that *H* and *D* now depend on the position of the shell via *θ* and *ϕ*. However, for conciseness, we will avoid writing explicitly the dependence. The Gauss constraint requires

4.3 $$K=H^{2}-D^{2}=1,$$

which we satisfy by setting $H=\sqrt{1+D^{2}}$. This allows us to write the bending energy in the simple form

4.4 $$E_{b}=\frac{Et^{3}}{12(1-\nu^{2})}\int \left[(\nu +1){\left(\bar{\kappa }-\sqrt{1+D^{2}}\right)}^{2}-(\nu -1)D^{2}\right]dA.$$

Note that such a simple form is only possible when the preferred bend is isotropic, as it does not require us to specify the orientation of the achieved curvature frame with respect to the preferred one.

Before instability, the shell is spherical, so we have *D* = 0, and we may conduct the integral to find the energy is $E_{b}=(Et^{3}/12(1-\nu^{2}))(\nu +1){\left(\bar{\kappa }-1\right)}^{2}A$ where *A* is the area of the shell. If we now consider a small amplitude perturbation from this spherical state, giving rise to a small *D*(*θ*, *ϕ*) ≠ 0, the principal curvatures take the form

4.5 $$\kappa_{1}(\theta ,\phi )=1+D(\theta ,\phi )+H.O.T$$

and

4.6 $$\kappa_{2}(\theta ,\phi )=1-D(\theta ,\phi )+H.O.T,$$

Similarly, inserting *D* into the energy and expanding, we obtain

4.7 $$E_{b}=\frac{Et^{3}}{12(1-\nu^{2})}\int \left[(\nu +1){\left(\bar{\kappa }-1\right)}^{2}+(2-(1+\nu )\bar{\kappa })D^{2}+\frac{1}{4}(1+\nu )\bar{\kappa }D^{4}+\cdots \right]dA.$$

Crucially, the coefficient of the second-order term changes sign at the critical curvature load

4.8 $${\bar{\kappa }}_{c}=\frac{2}{1+\nu }.$$

Beyond this threshold, any perturbing function *D*(*θ*, *ϕ*) will save energy compared with the spherical state, so the system has become unstable. Looking at the fourth-order term, we see that beyond the threshold, we expect $|D|=\sqrt{\bar{\kappa }-{\bar{\kappa }}_{C}}$. Both these results make no assumptions about the depth of the shell but exactly coincide with the results obtained earlier for shallow shells.

Formally, the above argument is not quite sufficient to prove instability, as not every form of *D*(*θ*, *ϕ*) corresponds to a real isometry of the surface. The issue arises because, although we have ensured the Gauss equation is satisfied, in deep shells the curvatures must also satisfy the Codazzi–Mainardi compatibility equations to ensure they describe a real surface. However, fortunately, a buckling isometry does indeed exist, as was identified by Lord Rayleigh in the study of vibration modes of shells [51]. In the case of a sphere with a hole around the south pole, the first-order isometry is given by

4.9 $$\delta R=R\sum_{n}^{\infty }(n+\cos\theta )\tan{\left(\frac{1}{2}\theta \right)}^{n}(A_{n}\cos(n\phi )+B_{n}\sin(n\phi )),$$

4.10 $$\delta \phi =\sum_{n}^{\infty }\tan{\left(\frac{1}{2}\theta \right)}^{n}(B_{n}\cos(n\phi )-A_{n}\sin(n\phi ))$$

4.11 $$\mathrm{and}\;\;\;\;\;\delta \theta =-\sin\theta \sum_{n}^{\infty }\tan{\left(\frac{1}{2}\theta \right)}^{n}(A_{n}\cos(s\phi )+B_{n}\sin(n\phi )),$$

where *n* is an integer that corresponds to the mode number of the solution, while *A*_*s*_ and *B*_*s*_ are amplitudes that may be fixed to any small value. We also note that modes with *n* = 0, 1 are solid body motion and rotation, respectively, with no deformation. However, every mode with *n* ≥ 2 gives *D* ≠ 0 and hence becomes unstable at our buckling threshold. In figure 5*b* we show the folding deformation induced by the mode *n* = 2 isometry. A direct computation reveals that the *n* = 2 mode indeed achieves the lowest energy of all the pure modes, in agreement with numerical and experimental observations.

A detailed numerical and experimental study of these curvature-induced instabilities in bilayer spherical shells was recently presented by Pezzulla *et al*. [40]. The authors use a combination of theoretical arguments and fitting to propose that the folding of a spherical cap with angular size *θ*_0_ occurs at the threshold curvature

$$\bar{\kappa }R=\frac{a}{\theta_{0}^{2}}\left(\frac{t}{R}\right)-c-\frac{b}{\pi -\theta_{0}}\sqrt{\frac{t}{R}},$$

where the parameter $a=\sqrt{10+7\sqrt{2}}$ was found by matching the theory to the flat plate case, while *b* = 3.6 and *c* = −0.98 were found fitting experimental/numerical data with *ν* = 0.5. This result is more ambitious than the current work, as it aims to also capture the effect of boundary layer stretching, leading to a thickness-dependent result, whereas we focus on the truly thin isometric limit. However, taking this limit of their result, in which *t*/*R* vanishes, the predicted threshold would be $\bar{\kappa }=-c=0.98$, which contrasts with ours at $\bar{\kappa }R=4/3\approx 1.33$. In figure 5*c*, we compare our result with the thinnest data available in [40] (*t*/*R* = 10^−3^) for a range of angular depths of shell. We see an extremely encouraging level of agreement, particularly for the deeper shells. Agreement for shallower shells is actually somewhat worse because these shells enter a regime where the boundary layer extent, $\sqrt{Rt}$, covers a large fraction of the shell. We thus see that the shallow theory actually applies in a limit where the shell is geometrically shallow, but large enough and thin enough so that the boundary layer is still negligible.

## 5. Summary and conclusion

In this paper, we have studied a simple model of instabilities in active shells that arise from the geometric incompatibility of their intrinsic and extrinsic curvatures. We have proposed a simple model, based on the requirements that the shell: (1) be geometrically shallow, (2) be thin enough to only deform isometrically, (3) has metric encoding homogeneous Gauss curvature *K*, and (4) is subject to thickness variations that encode a homogeneous preferred curvature tensor $\bar{\kappa }$. If these conditions are met, the configuration of the shell is simply given by the minimization of the bending energy over achieved homogeneous curvature κ, subject to the constraint that the achieved Gauss curvature is *K*.

**Figure 6.**
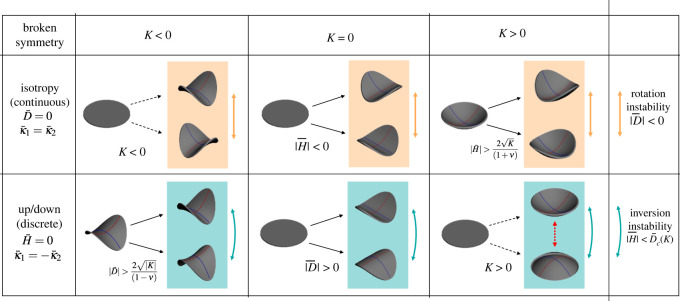
Summary of curvature-induced instabilities in thin shallow shells with different Gauss curvature. Top row: shells with isotropic preferred curvature, $\bar{D}=0$, break isotropy, making rotationally degenerate states (two shown) that are continuously connected. Isotropy is broken by all *K* < 0 shells but only past a threshold $\bar{H}$ for *K* > 0. Yellow arrows mark the associated *rotation* instability (between degenerate states) associated with traversing the $\bar{D}=0$ line, with vanishing threshold, $\bar{D}=0^{\pm }$. Second row: shells with up/down symmetric preferred curvature, $\bar{H}=0$, break this discrete symmetry’ leading to two equivalent states and bistability. Up/down symmetry is broken by all *K* > 0 shells but past a threshold $\bar{D}$ for *K* < 0. The *inversion* instability, marked by the curved cyan arrows, is associated with exiting the bistable region and jumping to an inverted state. The finite thresholds for inversion are equation (3.8) for *K* = 0 and equation (3.23) for *K* < 0, while for *K* > 0 inversion requires a non-isometric pathway, leading to threshold that diverges in the thin limit.

The resultant model has three cases delineated by Gauss curvature, *K* < 0, *K* = 0 and *K* > 0. In each case, we have given a full phase diagram showing the achieved state of the shell as a function of the preferred curvature. The phase diagrams reveal three types of curvature-driven instabilities, in which the principal achieved curvatures of the shell *rotate*, *symmetry-break* and *invert*, as summarized in figure 6. The model predicts modest and thickness-independent threshold curvatures $\propto \sqrt{K}$ for all the instabilities, except inversion in the *K* > 0 case, which cannot proceed isometrically and hence has a thickness-dependent threshold that diverges in the thin limit and is beyond the reach of the simple model.

*Symmetry-breaking* transitions arise in two flavours. In one case, we have isotropic preferred curvature $\bar{\kappa_{1}}=\bar{\kappa_{2}}$ ($\bar{D}=0$), which is larger than permitted by the Gauss constraint, *κ*_1_*κ*_2_ = *K*. It eventually becomes favourable for one achieved curvature to grow at the expense of the other, to better fit the preferred curvature while maintaining *K*. For *K* = 0, this occurs for any finite preferred curvature and the system rolls up in an arbitrary direction, while for *K* > 0 this involves a spherical cap folding along an arbitrary direction and occurs supercritically past a threshold preferred curvature. For *K* < 0, isotropy is always broken by the shell due to the Gauss requirement to form a saddle, even when the preferred curvature is actually zero, so this type of instability is not observed by changing preferred curvatures at fixed *K* < 0. However, the symmetry-broken state is present and the instability can be observed by an active change in the metric from flat to *K* < 0 [39], in which case the saddle must ‘decide’ an orientation as it forms. Since this symmetry-breaking is associated with breaking of a continuous (rotationally invariant) symmetry, we call it a *continuous symmetry-breaking* instability.

The second *symmetry-breaking* case arises with equal and opposite preferred curvatures, $\bar{\kappa_{1}}=-\bar{\kappa_{2}}$ ($\bar{H}=0$), encoding a preferred symmetric saddle. In the *K* = 0 case, the sheet will roll up in one of these two ways, while for *K* < 0 it will form a symmetric saddle for low preferred curvatures and either of two asymmetric saddles beyond a threshold. The energetic motivation is the same as the continuous case, but this time a discrete up/down symmetry is broken, yielding bistability. We thus call it a *discrete symmetry-breaking* instability. In the *K* > 0 case, up/down symmetry is always broken by the Gauss requirement to have both curvatures with the same sign, but the instability can again be observed by an active change in the the metric from flat to *K* > 0 [39], in which case the cap must ‘decide’ whether to pop up or down as it forms. Curiously, breaking isotropy for *K* > 0 and breaking up/down symmetry for *K* < 0 are described by almost identical threshold and amplitude equations, except with *D* and *H* as the order parameter, and $\bar{H}$ and $\bar{D}$ as the control parameter.

The buckling threshold of *symmetry-breaking* instabilities in both negative and positive Gauss curved systems depends on the magnitude of the preferred curvature load relative to that of the Gauss curvature. Throughout, we have discussed instabilities in terms of changing extrinsic preferred curvature, ${\bar{\kappa }}_{1}$ and ${\bar{\kappa }}_{2}$, at constant *K*, indicating active shape changes that vary through the thickness. However, one may achieve the same ends by varying *K* at fixed ${\bar{\kappa }}_{1}$ and ${\bar{\kappa }}_{2}$, indicating active shape changes to the metric. In such a case, the shell will move along a diagonal line radiating out from the origin on any of the phase diagrams, which suffices to trigger either symmetry-breaking transition by moving along the $\bar{D}=0$ and $\bar{H}=0$ axes.

The *rotation* instabilities arise as a consequence of states that break isotropy. This angular symmetry-breaking leads to Mexican-hat-style energies in which the curvature frame may rotate without penalty. Applying a small bias to such a potential, by traversing the $\bar{D}=0$ line, will break the degeneracy and produce a large rotation without an energy change. All three Gauss cases have such states and hence show rotation instabilities. Finally, the *inversion* instabilities arise from changing the sign of $\bar{H}$ to apply an up/down bias to the bistability generated by the *discrete symmetry-breaking* transitions. In this case, a finite amount of $\bar{H}$ is required to eliminate a minimum, leading to a subcritical inversion of the shell at the limit of bistability. For *K* < 0 cases, this occurs at a modest thickness-independent threshold, while for *K* > 0 it does not, so the instability is not present in the model, though it does occur in real shells via boundary layer stretching [40,50].

Rotation and inversion instabilities are not naturally triggered by manipulating *K* rather than $\bar{D}$ and $\bar{H}$, as they occur by crossing the $\bar{D}=0$ and $\bar{H}=0$ axes on the phase diagram, which no line radiating from the origin will do. However, for *K* < 0, if a shell is in the local minimum of the bistable region, it can exit the bistable region and invert via changes in *K*, as illustrated by the grey line in the phase diagram of figure 4.

The omission of stretch effects and boundary layer mechanics underpins the simplicity of our model, but is also its key limitation. Although the boundary layer may be safely neglected in suitably thin shells, where the extent of the boundary layer, $\propto \sqrt{t}$, is negligible compared with the shell’s extent, the boundary layer is of considerable importance in many physical shells. Firstly, the boundary layer permits the *K* > 0 inversion instability. Moreover, even in very thin shells, the boundary layer can allow the shape of the shell’s perimeter to couple its achieved curvature, particularly in situations of a continuous broken symmetry; for example, a Gauss flat bilayer square always rolls up along a diagonal [34,45,46], rather than being truly degenerate. In modest thickness shells, the boundary layer can also nucleate or delay even those instabilities that could proceed isometrically [40,46]. However, the simple isometric model nevertheless gives considerable insight into why these instabilities occur and when the boundary layer is a complication rather than an essential ingredient. It also clarifies when we should expect thickness-independent thresholds and is asymptotically correct at small thickness.

By contrast, the shallowness assumption in our model does not appear to be a major limitation. Indeed, we have shown that the threshold for the symmetry-breaking folding of a spherical cap subject to excessive preferred curvature is also correct for a deep spherical shell. Here, the Rayleigh isometry of the sphere potentially allows a much deeper and richer exploration of the response of such a shell to excessive curvature, which we reserve for future work.

## Data Availability

This article does not contain any additional data.
